# Regulatory role of hexosamine biosynthetic pathway on hepatic cancer stem cell marker CD133 under low glucose conditions

**DOI:** 10.1038/srep21184

**Published:** 2016-02-16

**Authors:** Shu-Hai Lin, Tengfei Liu, Xiaoyan Ming, Zhi Tang, Li Fu, Philippe Schmitt-Kopplin, Basem Kanawati, Xin-Yuan Guan, Zongwei Cai

**Affiliations:** 1 State Key Laboratory of Environmental and Biological Analysis, Department of Chemistry, Hong Kong Baptist University, Hong Kong SAR; 2 Department of Clinical Oncology, Li Ka Shing Faculty of Medicine, the University of Hong Kong, Hong Kong SAR; 3 Shenzhen University Cancer Center, Shenzhen, 518060, China; 4 Helmholtz-Zentrum Muenchen-German Research Center for Environmental Health, Research Unit of analytical Biogeochemistry, Ingolstaedter Landstrasse 1, D-85764 Oberschleißheim, Germany

## Abstract

Cancer was hypothesized to be driven by cancer stem cells (CSCs), but the metabolic determinants of CSC-like phenotype still remain elusive. Here, we present that hexosamine biosynthetic pathway (HBP) at least in part rescues cancer cell fate with inactivation of glycolysis. Firstly, metabolomic analysis profiled cellular metabolome in CSCs of hepatocellular carcinoma using CD133 cell-surface marker. The metabolic signatures of CD133-positive subpopulation compared to CD133-negative cells highlighted HBP as one of the distinct metabolic pathways, prompting us to uncover the role of HBP in maintenance of CSC-like phenotype. To address this, CSC-like phenotypes and cell survival were investigated in cancer cells under low glucose conditions. As a result, HBP inhibitor azaserine reduced CD133-positive subpopulation and CD133 expression under high glucose condition. Furthermore, treatment of N-Acetylglucosamine in part restores CD133-positive subpopulation when either 2.5 mM glucose in culture media or glycolytic inhibitor 2-deoxy-D-glucose in HCC cell lines was applied, enhancing CD133 expression as well as promoting cancer cell survival. Together, HBP might be a key metabolic determinant in the functions of hepatic CSC marker CD133.

Mounting evidence demonstrates that tumor is hierarchically organized and consists of heterogeneous population of cells, in which a small subpopulation of cells named cancer stem cells (CSCs), has been best characterized by the features to initiate tumor growth, self-renew, and differentiate^1^. Up to date, CSCs have been found in a wide range of human cancers, including liver cancer^2^, breast cancer^3^, glioblastoma^4^, lung cancer^5^, and leukemia^6^.

CD133 was first identified as a primitive hematopoietic and progenitor cell’s marker and widely used alone or in combination with other stem cell markers to enrich stem cell from multiple tissues like brain, liver, pancreas and bone marrow^7^. It’s now widely used as a CSC marker to isolate CSCs from human liver tumors. It’s true and clear that CD133-positive, compared to the counterpart CD133-negative, endows with the features, the preferential capacities of cancer cell and stem cell, of increased ability to promote tumorigenicity and metastasis *in vitro* and *in vivo*, as well as the ability to differentiate into varied lineages. CD133-positive subset harbors the upregulation of stem cell related genes, such as Bmi1, Notch, and beta-catenine. Moreover, CD133-positive subpopulation cells also show the augment ability to form undifferentiated spheroids primarily as well as *in vitro* passages, meantime with upregulation of stem cell associated genes^1^. Aberrantly preferential upregulation of Akt and Bcl-2 confers the CD133-positive subpopulation cells with survival characteristics, leading to chemoresistance and radioresistance^8^. Recently, Chai *et al.* reported that miR-142–3p regulates CSC-like properties in hepatocellular carcinoma (HCC) via the direct targeting of CD133^9^. More interestingly, the posttranslational modifications including phosphorylation and N-linked glycosylation of CD133 are also associated with CSC-like phenotypes and tumor growth^10^,^11^.

Although the growing evidence elucidates some integral molecules and signaling pathways responsible for the stemness maintenance and differentiation of CD133-positive subpopulation cells^12^,^13^,^14^, the metabolic features of CD133-positive enriched CSCs and the effects of nutrients in CSCs remain inconclusive. Recently, energy metabolism in cancer cells has been highlighted in the tumor initiation and development and Warburg effect has been re-surveyed worldwidely^15^.

However, metabolic rewiring in CSCs has not been well characterized. To address this issue, it would be necessary to perform the emerging technique metabolomics for cellular metabolome. In this study, we isolated CD133-positive and counterpart CD133-negative subsets from human liver cancer cell line PLC8024 followed by metabolomics study based on Fourier transform ion cyclotron resonance mass spectrometry (FTICRMS) and ultrahigh-performance liquid chromatography quadrupole time-of-flight mass spectrometry (UPLC-QTOFMS) to profile the cellular metabolome. Uridine diphosphate-N-acetylglucosamine (UDP-GlcNAc), an end product in hexosamine biosynthetic pathway (HBP) was determined as the elevated level in CD133-positive subsets. This finding prompted us to ask whether HBP impacts CSC-like phenotype. Our data indicated that inhibition of HBP pathway by targeting the limiting-rate enzyme resulted in lowering the percentage of CD133-positive subpopulations. Furthermore, N-acetylglucosamine (GlcNAc), which has been widely used to increase HBP product, could in part restore CD133-positive CSC phenotype and promote cell survival under low glucose conditions. Collectively, our investigation demonstrated that HBP might coordinate with glycolytic pathway for the regulation of CD133 in hepatoma carcinoma cell lines.

## Results

### Metabolomics identifies hexosamine biosynthetic pathway in CD133-positive cancer cells

**Figure 1:**
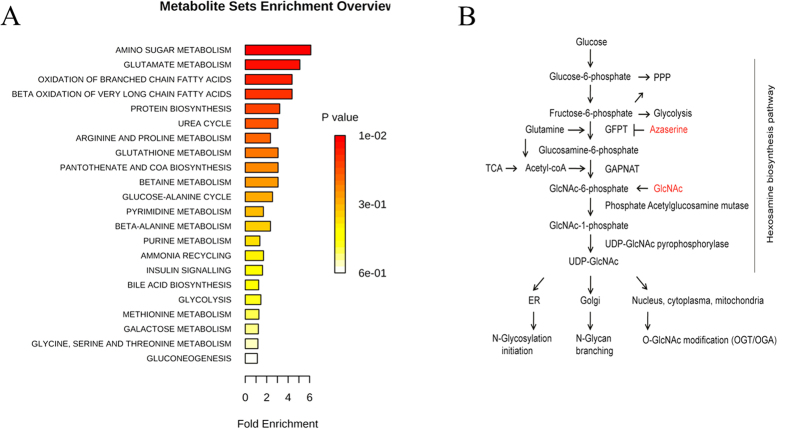
Hexosamine biosynthetic pathway (HBP) was highlighted in cancer stem cells. (**A**) Metabolite sets enrichment analysis of the differential metabolites reveals that the highest impact of the pathways is amino sugar metabolism. (**B**) Schematic diagram of HBP. GFPT is the first rate-limiting enzyme in HBP, and N-acetyl-glucosamine (GlcNAc) can enter into and activate the HBP. The proposed role of HBP in maintaining the cancer stem cell phenotype enriched by CD133 in hepatoma carcinoma cell lines for further investigations.

The CD133-positive subpopulation percentage of HCC cell lines have been examined and reported previously^16^. In our study, high resolution direct-infusion ion cyclotron resonance Fourier-transform mass spectrometry (DI-ICR-FT-MS) and ultrahigh performance liquid chromatography quadrupole time-of-flight mass spectrometry (UPLC-QTOFMS) were employed for untargeted metabolomics. The representative mass spectrum is shown in Figure S1. In the metabolomics workflow, peak picking followed by feature selection was performed by intensities of ions using XCMS package in R language. The metabolite identification was conducted by high mass accuracy and fragment ions as well as reference standards. As a result, a total of 32 differential metabolites were identified, covering glucose metabolism, amino acid metabolism, fatty acid and lipid metabolism (Table 1). Metabolite sets enrichment analysis (MSEA) was performed to highlight the metabolic difference between CD133-positive and CD133-negative subpopulations of PLC8024 cell line. Amino sugar metabolism, also known as hexosamine biosynthetic pathway (HBP), was identified to be most significant (Fig. 1A). Amino sugars are physiologically synthesized through the hexosamine biosynthetic pathway. In this pathway, the rate-limiting enzyme glutamine : fructose-6-phosphate-amidotransferase (GFPT) catalyzes fructose-6-phosphate to form glucosamine-6-phosphate (GlcN6P) by consuming glutamine as an amino donor^17^. GlcN6P is rapidly converted to the end product UDP-GlcNAc which provides amino sugars for the biosynthesis of N- and O-linked protein glycosylation (Fig. 1B). In this regard, our metabolomic data raised a question whether HBP plays a key role for discriminating CD133-positive stemness phenotype in cancer cells.

### Inhibition of HBP decrease the percentage of CD133-positive in HCC cell lines

**Figure 2:**
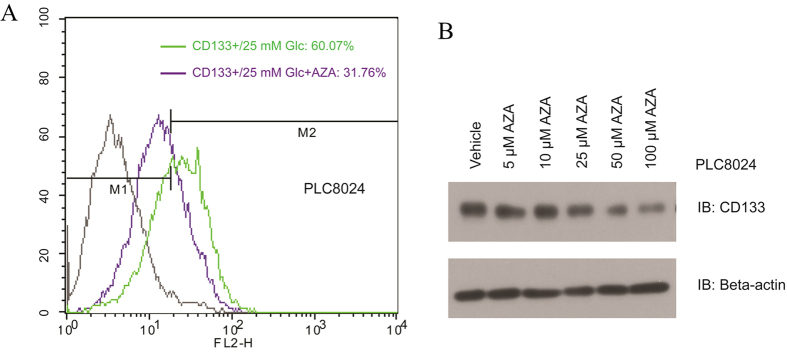
Hexsoamine biosynthetic pathway involved in the maintenance of CSC-like phenotype in PLC8024 cell line. (**A**) AZA treatment reduces the percentage of CD133 in PLC8024 parental cells. (**B**) Expression of CD133 was determined by western blotting. The PLC8024 was cultured in high glucose medium in presence of AZA with different concentrations (5, 10, 25, 50, 100 μM ) or a vehicle control (DMEM only). Beta-actin was used as loading control.

Before verifying the role of HBP for maintaining the CSC-like phenotypes, we measured SOX2 and found higher expression in CD133-positive subpopulation compared to CD133-negative cells (Figure S2), indicating that CD133 is a good marker of stemness^18^. Therefore, we evaluated the next examination by using the marker CD133 and/or the percentage of CD133-positive cells. Firstly, we introduced an inhibitor of HBP, azaserine (AZA), which is widely used as a glutamine analog and the inhibitor of GFPT. We observed that the proportion of CD133-positive subpopulation declined from 60.07% to 31.76% upon AZA treatment (Fig. 2A) and Western blotting assay also revealed that CD133 expression level negatively correlated with the dosage of AZA in PLC8024 cell line (Fig. 2B).

### Glycolysis involved in the maintenance of CD133-positive subsets in HCC cell lines

**Figure 3:**
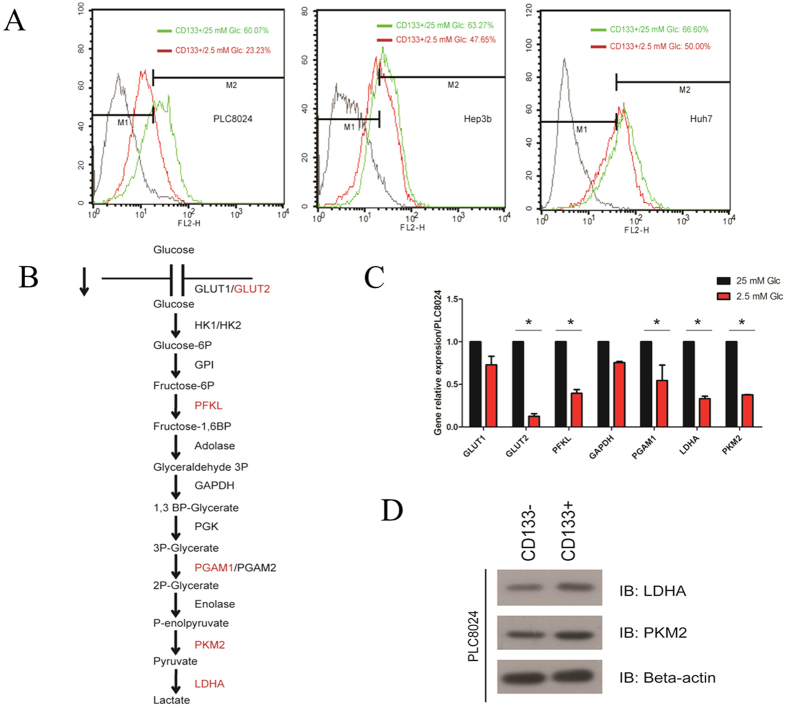
Glucose plays an important role in maintaining the cancer stem cell phenotype enriched by CD133 in hepatoma carcinoma cell lines. (**A**) The percentage of CD133-positive subsets was analyzed by flow cytometry. The green line represent the cell were maintained in high-glucose (25 mM) condition and red line indicate the cell were cultured in low glucose (2.5 mM) condition. (**B**) Schematic diagram of glycolytic pathway and involved genes. (**C**) Real-time PCR analysis of mRNA expression of GLUT1, GLUT2, PFKL, GAPDH, PGAM1, LDHA and PKM2 under high and low glucose conditions in PLC8024 cell line. (**D**) The key glycolytic enzymes (LDHA and PKM2) were determined by Western blotting. Beta-actin was used as loading control.

Thus far, glucose has been well known to serve as one of the most important carbon sources and plays a critical role in highly proliferative cancer cells, although not all kinds of cancers. To examine the effect of glucose concentration on CSC-like phenotype, we further utilized low glucose condition (2.5 mM glucose in culture medium) for three HCC cell lines PLC8024, Huh7 and Hep3B containing CD133-positive subpopulation. The percentages of CD133-positive subset decreased significantly compared to the normal culture medium with high glucose (25 mM glucose) as the control: the percentage of CD133-positive subpopulation declined from around 60% to 23.23% in PLC8024, and the percentages decreased from 63.27% to 47.65% and 66.60% to 50% in Hep3B and Huh7 cell lines, respectively (Fig. 3A).

**Figure 4:**
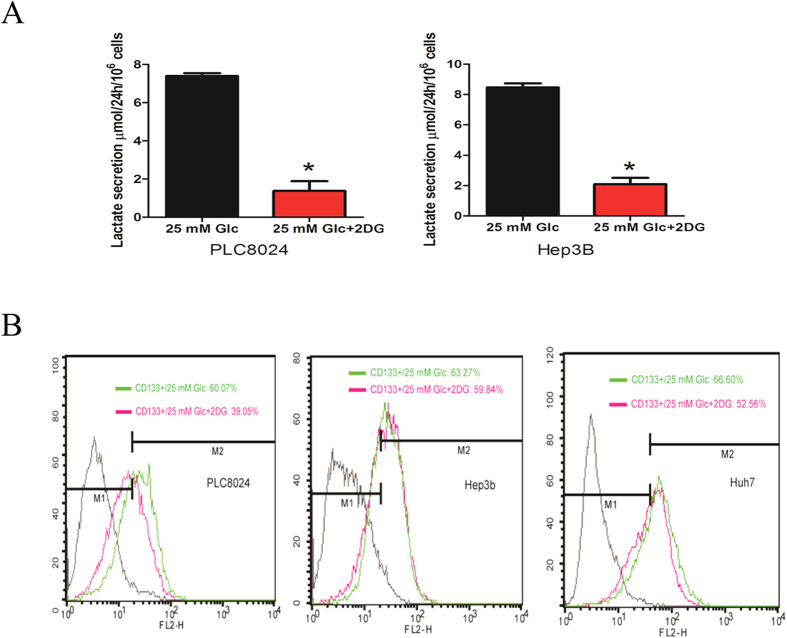
(**A**) Lactate was measured fluorometrically. Cells were seeded and serum starved 16 h followed by replaced with fresh medium in presence of 20 mM 2-DG or not. Lactate measured in cell culture supernatants showing quantity (μmol) per 10^6^ of tested cells. Samples were diluted 2–5 fold. (**B**) HCC cell lines were cultured under high glucose medium in presence of 20 mM 2-DG or vehicle control (DMEM only) for 16 h, and CD133-positive phenotype were detected by flow-cytometry. Each experiment was repeated at least three times. Representative data are shown.

Furthermore, we examined the gene expression pattern of the glycolytic pathway under low glucose condition by using the quantitative RT-PCR and observed a list of vital genes including GLUT2, PFKL, PGAM1, PKM2 and LDHA (Fig. 3B,C) were consistently down-regulated significantly, suggesting that the glycolytic pathway might play a vital role for the maintenance of CSC-like phenotype. To confirm the CSC-like phenotype, the fluorescence-activation cell sorting (FACS) technique was used to isolate the CD133-positive subpopulation cells from PLC8024 and Western blotting was also performed to confirm subsequently that the expressions of PKM2 and LDHA (Fig. 3D). These two enzymes at the last step of glycolytic pathway were also upregulated moderately, hinting the glycolytic activation in CSCs of HCC cell line. To further validate the CSC-like phenotype is glycolytic pathway-dependent, we treated the cells with 2-DG, an inhibitor of glycolytic pathway. 2-DG is the glucose homologues and can be transported into the cells whereas could not be metabolized downstream, resulting in inhibition of glycolytic bioprocess. After 16h-administration of the HCC cell lines with 2-DG, the lactate secretion was significantly inhibited in PLC8024 and Hep3B cell lines (Fig. 4A). The flow-cytometry assay displayed that the CD133-positive subpopulation cells was substantially reduced in PLC8024 (Fig. 4B). All the data suggested that glycolytic pathway played a critical role in regulating the CSCs enriched by CD133 in HCC cell line PLC8024, although only slight decrease of CD133-positive subpopulation in Hep3B and Huh7 cell lines was observed.

### GlcNAc involved in the regulation of CD133-positive subset under glucose deprivation

**Figure 5:**
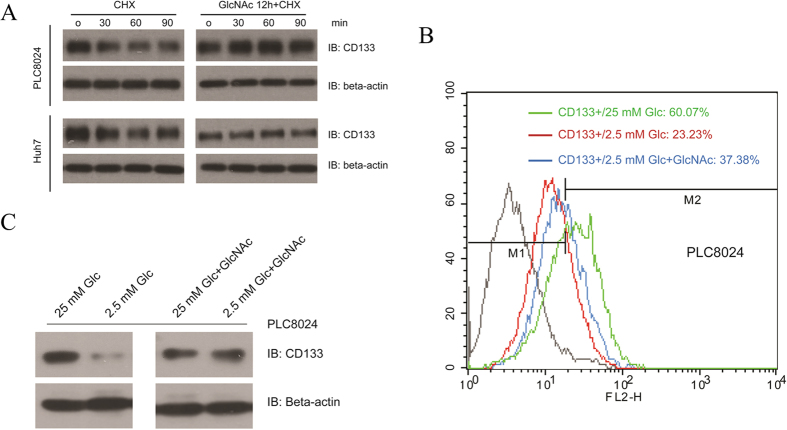
The supplementation of GlcNAc plays an important role in maintenance of cancer stem cell-like phenotype. (**A**) PLC8024 and Huh7 cell lines were pre-treated with 10 mM GlcNAc or the vehicle control (DMEM only) for 12 h. After that, cells were treated with CHX (50 μg mL^−1^) or the vehicle control (DMSO) for indicated time. The expression of CD133 was examined by Western blotting and Beta-actin was used as loading control. (**B**) The PLC8024 cell line was cultured in high glucose, low glucose or low glucose in presence of 10 mM GlcNAc for 48 h and CD133-positive subpopulation was determined by flow cytometry. (**C**) The PLC8024 cells were cultured in high glucose, low glucose or low glucose in presence of 10 mM GlcNAc for 48 h and expression of CD133 was examined by Western blotting and Beta-actin was used as loading control.

**Figure 6:**
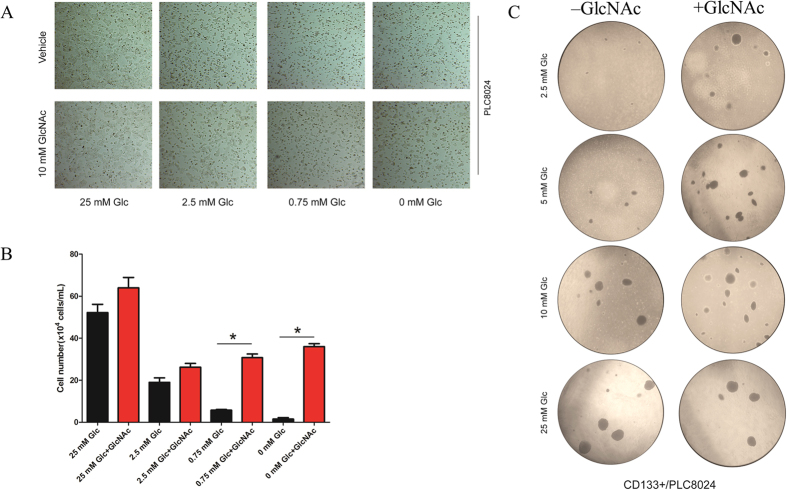
The supplementation of GlcNAc plays an important role in cancer cell survival under low glucose condition. (**A**) PLC8024 cells were seeded in 6-well plate and maintained in conditional medium with indicated glucose concentration (25 mM, 2.5 mM, 0.75 mM and 0 mM) in presence of 10 mM GlcNAc or the vehicle control (DMEM only) for 48 h. The representative images were captured by bright field microscope. (**B**) The histogram represents quantitative analysis of the mean values of survival cells in triplicate determinations. Asterisk indicates the significant difference. *P  0.05. (**C**) The conditional soft agar assay medium with different glucose concentration (25 mM, 10 mM, 5 mM, 2.5 mM) were pre-prepared. The CD133-positive subpopulation cells and CD133-negative counterpart isolated from PLC8024 were maintained in conditional soft agar assay medium with additional of 10 mM GlcNAc or the vehicle control (DMEM only) for 3 weeks. The representative images were captured by bright field microscope. All pictures were all at same magnification, resolution and size. The histogram represents the quantitative analysis of mean values of colony formation in conditional soft agar assay medium in triplicate determinations. Asterisk indicates the significant difference. *P  0.05.

For a better understanding of coordination of glycolysis and HBP in maintenance of CSC-like phenotype, we further examined the role of HBP pathway in the regulation of CD133-positive subpopulation. It’s well documented that GlcNAc could enter into the HBP and increase the production of UDP-GlcNAc, resulting in activation of HBP^19^. In light of this, we maintained the cell in presence of 10 mM GlcNAc overnight, after that, we treated the HCC cell lines with 50 μg mL^−1^ Cycloheximide (CHX), an inhibitor of protein biosynthesis bioprocess. Western blotting indicated that the GlcNAc could elevate the protein stability (Fig. 5A). In addition, we found GlcNAc could restore the proportion of CD133-positive subpopulation under glucose deprivation partially but obviously, the proportion of CD133-positive subset was recovered to 37.38% after additional of 10 mM GlcNAc compared to 23.23% under 2.5 mM glucose conditional medium (Fig. 5B). Consistently, Western blotting also showed that GlcNAc could restore the CD133 protein expression under glucose deprivation conditional culture medium obviously (Fig. 5C). Intriguingly, optical microscope imaging demonstrated that the GlcNAc could maintain the cell growth, even under extremely low glucose condition or withdrawal of glucose completely (Fig. 6A,B). All of these data confirmed that the GlcNAc could support cell survival under glucose deprivation, maintaining the CSC-like phenotype. To validate our hypothesis and previous finding, we introduced the soft-agar assay, which is the *in vitro* experiment to evaluate the long term proliferation and self-renewal ability of CSCs. After isolation the CD133-positive subset and negative counterpart via FACS technique, we kept these cells in soft agar with the different glucose concentration from low to high, in presence of GlcNAc or not. After 3-week culture, we found that GlcNAc could promote the colony formation under low glucose concentration significantly (Fig. 6C).

## Discussion

Metabolic reprogramming has been recognized as a hallmark in cancer cells^15^. In particular, Warburg effect has been hypothesized many decades ago to emphasize the role of glucose metabolism in tumorigenesis^20^,^21^. The branched pathways of glycolysis in cancer cells, however, are still ill defined. In this regard, it is necessary to apply a new approach for a broader investigation of metabolic pathways. Metabolomics as the downstream of systems biology, by using an array of chromatographic techniques coupled with mass spectrometry, has been developed and applied in cellular metabolism. To characterize the metabolic signatures in CSCs, both DI-ICR-FT-MS and UPLC-QTOFMS were used in non-targeted metabolomics to obtain high-resolution snapshots of the metabolic state of a system, by providing highly accurate masses for metabolite identification.

Of note, HBP was identified to be one of important pathways for discrimination of CSCs and non-CSCs using CD133 as a marker in our study, suggesting that HBP may play a critical role in maintenance of stem cell-like phenotype. HBP diverts from F6P, requiring glutamine for incorporation of amino group in the downstream products. The critical role of HBP has been demonstrated to provide glycosylation of proteins including O-GlcNAcylation, promoting tumor cell growth under glucose deprivation^22^,^23^. In this work, we observed that inhibition of HBP leaded to down-regulation of CD133-enriched CSCs, suggesting that HBP involved in the maintenance of CSC-like phenotype within HCC cell line and corroborating our metabolic characterization.

In order to modulate the HBP activity, we further administrated the cells with GlcNAc. It’s well documented that GlcNAc can enter into the cell via cell bulk endocytosis and is phosphorylated by the salvage pathway enzyme N-acetylglucosamine kinase. GlcNAc is converted to the intermediate metabolite GlcN6P and consequently contribute to the end production of the HBP, UDP-GlcNAc^19^,^24^. Our experiment data demonstrated that GlcNAc supplementation contributes to stabilize CD133 protein level, and promote cell survival. Recent published investigation demonstrate that CD133, beyond as a CSC marker, still functions involved in survival through regulation of autophagy and glucose uptake, which may be necessary for CSCs to survive in tumor microenvironment^25^,^26^. Whether HBP plays a role in maintaining CSC-like phenotype for cancer survival under glucose deprivation that common phenomenon observed in solid tumor, remain elusive and need to further study.

Inhibition of glycolytic pathway by 2-DG resulted in lower of CD133-positive phenotype. Unlike PLC8024 that with considerable downregulation, Huh7 and Hep3B cell lines showed not significant but reproducible decrease of CD133-positive subpopulation (Figure S3). We suspected that CD133-enriched CSCs within PLC8024 may be more susceptible to glucose metabolism modulation. Consistently and as we expected, when Huh7 and Hep3B were exposed to 2-DG in the presence of GlcNAc, we could observe slight but reproducible restoration of CD133-positive subgroups.

Nevertheless, our study raised more interest in the role of metabolism in CSCs. Indeed, a new item “metabostemness” was coined to describe the metabolic alterations in CSCs^27^. How metabolism impacts on stem cell-like phenotype shows potential interest and provides a new vista for cancer therapy^28^,^29^. Recently investigation indicated that levels of enzymes and metabolites involved in glycolysis, the citric acid (TCA) cycle, and cysteine and methionine metabolism are altered in colorectal CSCs. The metabolic signature provides mechanistic insights into colorectal CSCs phenotypes and may serve as potential biomarkers and therapeutic targets for future colorectal cancer treatment^30^. Our study showed significant description that HBP play a vital role in maintenance of CSCs’ phenotype within HCC, providing a new avenue for future CSCs-targeted cancer study.

## Methods

### Cell culture

PLC8024, Huh7 and Hep3B cell lines were obtained from the Institute of Virology, Chinese Academy of Medical Sciences (Beijing, China). All the cell lines were maintained in Dulbecco’s modified Eagle’s medium supplemented with 10% fetal bovine serum. All cells were cultured at 37 °C with an atmosphere of 5% CO_2_ and 95% air.

### Metabolomic profiling

CD133-positive CSCs were obtained by cell sorting from PLC8024, following by metabolite extraction and mass spectrometry-based metabolomic analysis. Direct-infusion ion-cyclotron-resonance Fourier-transform spectrometry (DI-ICR-FT-MS) was used for measurement of the metabolites, and home-made matrix generator was performed for peak alignment^31^. Meanwhile, ultrahigh performance liquid chromatography quadrupole time-of-flight mass spectrometry (UPLC-QTOFMS) was also employed combining with XCMS program in R language. Subsequently, the differentiating ions were introduced into the databases for metabolite identification, like massTRIX (www.masstrix.org) and Metlin (http://metlin.scripps.edu). As a result, the compilation of differentiating metabolites was assigned to metabolic pathways for discriminating CD133-positive cells from CD133-negative subpopulations. The details of metabolite extraction and mass spectrometric conditions and statistical analysis were described in supporting information.

### Reagents and antibodies

Mouse IgG1 K Isotype Control PE was purchased from eBioscience (San Diego, CA); anti-CD133/1 (W6B3C1) mouse mAb and phycoerythrin (PE)-conjugated CD133/1(AC133-PE) antibody were obtained from miltenyi (Aubum, CA) were obtained from Cell Signaling Technology (Beverly, MA); glycolysis related antibodies including anti-PKM2 (D78A4) rabbit mAb and anti-LDHA (C4B5) rabbit mAb from Glycolysis Antibody Sampler Kit (#8337) were purchased from Cell Signaling Technology (Beverly, MA); anti-Beta-actin (ab6276) mouse mAb were from Abcam (Cambridge, UK); horseradish peroxidase-conjugated goat anti-mouse and goat anti-rabbit were from Sigma – Aldrich (St. Louis, MO). Chemicals including 2-Deoxy-D-glucose (2DG), N-Acetylglucosamine (GlcNAc), Azaserine and Dulbecco′s Modified Eagle′s Medium powder (D5030) were purchased from Sigma–Aldrich (St. Louis, MO).

### Fluorescence-activated cell sorting

(PE)-conjugated CD133/1(AC133-PE) antibody was used for cell staining according to the manufacture’s protocol. Briefly, 1 × 10^7^ cells were determined and washed with sterile PBS twice. Resuspended cells in 80 μL buffer (PBS with 2% fetal bovine serum), additional of 20 μL FcR Blocking reagent followed with 10 μL CD133/1(AC133) antibody. Mixed gently and incubated at 4°C in the dark for 30 min. After washing for 3 times with PBS, cells were sorted and analyzed on a BD FACSVantage SE (BD Biosciences). Isotype matched mouse IgG used for negative control.

### Western blotting analysis

Cells were lysated in a RIPA buffer (50 mM Tris pH = 7.6, 1% Triton, 150 mM NaCl, 2 mM EDTA, protein inhibitor mixture(Roche), 1 mM NaF, 1 mM Na_3_VO_4_). Equal amounts of proteins were separated by 10% or 12% SDS-PAGE followed by transferred to PVDF membrane. The membranes were blocked in 5% non-fat milk for 1 h at room temperature and incubated with primary antibody overnight at 4 °C. After washing 3 times the second day, the blots were incubated with HRP-conjugated anti-mouse or anti-rabbit secondary antibodies at room temperature for 1 h. The antibody signal will be visualized using an enhanced chemiluminescence system (Pierce Biotechnology).

### RNA isolation, reverse transcription and qRT-PCR

Cells were collected and washed with PBS twice, total RNA was extracted using Trizol Reagent (Takara Bio, Japan) and cDNA were synthesized using Prime Script^TM^ RT Master Mix (Takara, Japan) according to the manufacturers’ instructions, respectively. The quantitative Real-time PCR was carried out using FastStart Universal SYBR Green Master (Roche, Germany) and ABI PRISM 7900 Sequence Detector (Applied Biosystems, Foster City, CA). The cycling parameters were 95 °C for 30 min, 55 °C for 1 min, and 72 °C for 2 min for 40 cycles, followed by a melting curve analysis. The threshold cycle (CT) was measured during the exponential amplification phase, and the amplification plots were analyzed through SDS 1.9.1 software (Applied Biosystems, Foster, City, CA). The primers used for qRT-PCR listed in supplementary Table S1.

### Anchorage-independent growth Assay

Cells were trypsined and washed with PBS twice and were suspended in 0.3% soft agar dissolved with culture medium in 6-well plates at a density of 6000 cells per well. After 3 weeks, anchorage-independent colonies (≥50 cells) were counted under optical microscope in random 8 fields per well and photographed.

### Lactate production determination assay

Lactate production was measured using a lactate assay kit (BioVision) according to the manufacturer’s instructions as described otherwise^32^.

### Statistical analysis and metabolic annotation

Metabolomic data analysis followed our previous publications^33^,^34^. Briefly, the DI-ICR-FT-MS mass spectra were exported to peak lists for alignment. After performing this data filtration by 80% rule, two-tailed student’s t-test was used for calculation of *P*-value. In addition, the differential ions were implemented into MassTRIX interface (http://www.masstrix.org) to search for differentiating metabolites. The criteria in database searching were as follows: negative ionization (correct for H+ loss) was selected as scan mode; maximum error was up to 1.0 ppm. The acquired data from UPLC-QTOFMS were processed using XCMS package for a matrix containing arbitrarily assigned peak index (retention time-*m/z* pairs), sample names (observations) and ion intensity information (variables). Differential metabolites were interfaced with a web-based tool (http://www.metaboanalyst.ca/) for the pathway analysis and visualization. The Wilcoxon Mann−Whitney test was performed for the biochemical and metabolomic data, and *P* < 0.05 was considered as significant.

## Additional Information

**How to cite this article**: Lin, S.-H. *et al.* Regulatory role of hexosamine biosynthetic pathway on hepatic cancer stem cell marker CD133 under low glucose conditions. *Sci. Rep.* **6**, 21184; doi: 10.1038/srep21184 (2016).

## Supplementary Material

Supplementary Information

## Figures and Tables

**Table 1:** The metabolites in PLC8024 were putatively identified (CD133-positive versus CD133-negative).

Metabolite	raw mass (*m/z*)	Possible assignments	Fold-change	*P*-value	Postulated annotation
1	215.03281^(i−)^*	glucose	1.96↑	1.08E−05	glucose and glutamine anaplerosis
2	147.0766^(q + )^	glutamine	2.04↑	6.3E−04	
	145.0600^(q−)^				
3	148.0605^(q + )^	glutamate	1.46↑	1.7E−03	
	146.0439^(q−)^				
4	175.1193^(q + )^	L-Arginine	1.65↑	4.97E−04	amino acid metabolism
5	306.07653^(i−)^	Glutathione	1.70 ↑	3.74E−05	low levels of reactive oxygen species
	308.0912^(q + )^				
	306.0752^(q−)^				
6	321.0509^(q−)^	Thymidine monophosphate	2.65↑	3.19E−04	DNA synthesis
7	347.03988^(i−)^	Inosine 5′-monophosphate	1.58 ↓	1.27E−02	nucleotide synthesis
8	498.28959^(i−)^	Taurodeoxycholic acid	Unique in CD133+	1.72E−03	bile acid transportation
9	516.3014^(q+)^	Taurocholic acid	2.15↑	6.47E−05	
10	440.24617^(i+)^	Leukotriene E4	Unique in CD133+	6.57E−04	fatty acid biosynthesis
11	227.20167^(i−)^	Myristic acid	1.49↑	5.59E−04	
12	218.10340^(i−)^	Pantothenic Acid	1.73↑	1.31Eh03	Coenzyme-A synthesis
13	162.1107^(q+)^	L-Carnitine	1.30↑	1.22E−04	fatty acid beta-oxidation
14	204.1231^(q+)^	Acetylcarnitine	1.38↑	1.44E−03	
15	300.04910^(i−)^	N-Acetyl-D-glucosamine 6-phosphate/ N-Acetyl-D-glucosamine 1-phosphate	3.89↑	5.0E−02	hexosamine biosynthesis
16	606.07451^(i−)^	Uridine diphosphate-N-acetylglucosamine	4.50↑	1.2E−04	
17	399.14444^(i+)^	S-Adenosyl-L-methionine	1.67↑	8.10E−03	hypermethylation
18	261.10833^(i+)^	L-alpha-glutamyl-L-hydroxyproline	1.64↓	3.31E−03	Prolyl hydroxylase might be inhibited
19	132.0768^(q+)^	Creatine	1.62↑	7.31E−04	helps to supply energy
20	245.04318^(i−)^	Glycerophosphoglycerol	1.53↑	0.002.67	Lipid accumulation suggests lipogenesis with tumorigenesis, metastasis and stemness.
21	258.1101^(q+)^	Glycerophosphocholine	1.62↑	1.88E−03	
22	425.26622^(i+)^	1-heptadecanoyl-glycero-3-phosphate	2.06 ↑	8.79E−04	
23	518.3168^(q+)^	LysoPC(18:3)	2.34↑	5.35E−05	
24	520.3338^(q+)^	LysoPC(18:2)	1.85↑	4.15E−05	
25	524.3689^(q+)^	LysoPC(18:0)	1.71↑	4.64E−05	
26	548.3801^(q+)^	LysoPC(20:2)	2.44↑	1.19E−04	
27	648.3800^(q+)^	1-Palmitoyl-2-(5-keto-8-oxo-6-octenoyl)-sn-glycero-3-phosphatidylcholine	2.34↑	6.59E−05	
28	650.3959^(q+)^	1-Palmitoyl-2-(5-hydroxy-8-oxo-6-octenoyl)-sn-glycero-3-phosphatidylcholine	2.38↑	9.97E−07	
29	702.4985^(q+)^	PC(18:2/12:0)	2.76↑	4.065E−05	
30	764.5063^(q+)^	PC(22:6/13:0)	1.74↑	3.29E−04	
31	766.5308^(q+)^	PC(20:5/15:0)	1.86↑	8.61E−04	
32	780.5437^(q+)^	PC(22:4/14:1)	1.76↑	9.68E−04	

(i+): FTICRMS-ESI (+); (i−): FTICRMS-ESI (−); (q+): QTOFMS-ESI (+); (q−): QTOFMS-ESI (−).

^*^The mass was assigned to be [glucose+Cl]^−^; LysoPC: lysophosphatidylcholine; PC: phosphatidylcholine.

## References

[b1] Ma S. *et al.* Identification and characterization of tumorigenic liver cancer stem/progenitor cells. Gastroenterology 132, 2542–2556 (2007). 17570225 10.1053/j.gastro.2007.04.025

[b2] Suetsugu A. *et al.* Characterization of CD133 + hepatocellular carcinoma cells as cancer stem/progenitor cells. Biochem Biophys Res Commun 351, 820–824 (2006). 17097610 10.1016/j.bbrc.2006.10.128

[b3] Al-Hajj M., Wicha M.S., Benito-Hernandez A., Morrison S.J. & Clarke M.F. Prospective identification of tumorigenic breast cancer cells. Proc Natl Acad Sci USA 100, 3983–3988 (2003). 12629218 10.1073/pnas.0530291100 PMC153034

[b4] Singh S.K. *et al.* Identification of human brain tumour initiating cells. Nature 432, 396–401 (2004). 15549107 10.1038/nature03128

[b5] Kim C.F. *et al.* Identification of bronchioalveolar stem cells in normal lung and lung cancer. Cell 121, 823–835 (2005). 15960971 10.1016/j.cell.2005.03.032

[b6] Bonnet D. & Dick J.E. Human acute myeloid leukemia is organized as a hierarchy that originates from a primitive hematopoietic cell. Nat Med 3, 730–737 (1997). 9212098 10.1038/nm0797-730

[b7] Yin A.H. *et al.* AC133, a novel marker for human hematopoietic stem and progenitor cells. Blood 90, 5002–5012 (1997). 9389720

[b8] Ma S., Lee T.K., Zheng B.J., Chan K.W. & Guan X.Y. CD133 + HCC cancer stem cells confer chemoresistance by preferential expression of the Akt/PKB survival pathway. Oncogene 27, 1749–1758 (2008). 17891174 10.1038/sj.onc.1210811

[b9] Chai S. *et al.* Regulatory role of miR-142-3p on the functional hepatic cancer stem cell marker CD133. Oncotarget 5, 5725–5735 (2014). 25015418 10.18632/oncotarget.2167 PMC4170635

[b10] Wei Y. *et al.* Activation of PI3K/Akt pathway by CD133-p85 interaction promotes tumorigenic capacity of glioma stem cells. Proc Natl Acad Sci USA 110, 6829–6834 (2013). 23569237 10.1073/pnas.1217002110 PMC3637720

[b11] Liu Y. *et al.* Mutation of N-linked glycosylation at Asn548 in CD133 decreases its ability to promote hepatoma cell growth. Oncotarget 6, 20650–20660 (2015). 26029999 10.18632/oncotarget.4115 PMC4653032

[b12] Sakurai T., Maeda S., Chang L. & Karin M. Loss of hepatic NF-kappa B activity enhances chemical hepatocarcinogenesis through sustained c-Jun N-terminal kinase 1 activation. Proc Natl Acad Sci USA 103, 10544–10551 (2006). 16807293 10.1073/pnas.0603499103 PMC1502270

[b13] Yang Z. *et al.* Transient mTOR inhibition facilitates continuous growth of liver tumors by modulating the maintenance of CD133 + cell populations. PLoS One 6, e28405 (2011). 22145042 10.1371/journal.pone.0028405 PMC3228748

[b14] Zhang L. *et al.* BMP4 administration induces differentiation of CD133 + hepatic cancer stem cells, blocking their contributions to hepatocellular carcinoma. Cancer Res 72, 4276–4285 (2012). 22773665 10.1158/0008-5472.CAN-12-1013

[b15] Hanahan D. & Weinberg R.A. Hallmarks of cancer: the next generation. Cell 144, 646–674 (2011). 21376230 10.1016/j.cell.2011.02.013

[b16] Ma S. *et al.* Aldehyde dehydrogenase discriminates the CD133 liver cancer stem cell populations. Mol Cancer Res 6, 1146–1153 (2008). 18644979 10.1158/1541-7786.MCR-08-0035

[b17] Marshall S., Bacote V. & Traxinger R.R. Discovery of a metabolic pathway mediating glucose-induced desensitization of the glucose transport system. Role of hexosamine biosynthesis in the induction of insulin resistance. J Biol Chem 266, 4706–4712 (1991). 2002019

[b18] Kyjacova L. *et al.* Radiotherapy-induced plasticity of prostate cancer mobilizes stem-like non-adherent, Erk signaling-dependent cells. Cell Death Differ 22, 898–911 (2015). 25012501 10.1038/cdd.2014.97 PMC4423190

[b19] Wellen K.E. *et al.* The hexosamine biosynthetic pathway couples growth factor-induced glutamine uptake to glucose metabolism. Genes Dev 24, 2784–2799 (2010). 21106670 10.1101/gad.1985910 PMC3003197

[b20] Warburg O. On the origin of cancer cells. Science 123, 309–314 (1956). 13298683 10.1126/science.123.3191.309

[b21] Vander, Heiden M.G., Cantley L.C. & Thompson C.B. Understanding the Warburg effect: the metabolic requirements of cell proliferation. Science 324, 1029–1033 (2009). 19460998 10.1126/science.1160809 PMC2849637

[b22] Jones D.R. *et al.* The hexosamine biosynthesis pathway and O-GlcNAcylation maintain insulin-stimulated PI3K-PKB phosphorylation and tumour cell growth after short-term glucose deprivation. FEBS J 281, 3591–3608 (2014). 24938479 10.1111/febs.12879

[b23] Kang J.G. *et al.* O-GlcNAc protein modification in cancer cells increases in response to glucose deprivation through glycogen degradation. J Biol Chem 284, 34777–34784 (2009). 19833729 10.1074/jbc.M109.026351 PMC2787340

[b24] Lau K.S. & Dennis J.W. N-Glycans in cancer progression. Glycobiology 18, 750–760 (2008). 18701722 10.1093/glycob/cwn071

[b25] Chen H. *et al.* CD133/prominin-1-mediated autophagy and glucose uptake beneficial for hepatoma cell survival. PLoS One 8, e56878 (2013). 23437259 10.1371/journal.pone.0056878 PMC3577658

[b26] Chen H. *et al.* Low glucose promotes CD133mAb-elicited cell death via inhibition of autophagy in hepatocarcinoma cells. Cancer Lett 336, 204–212 (2013). 23652197 10.1016/j.canlet.2013.04.031

[b27] Menendez J.A. & Alarcon T. Metabostemness: a new cancer hallmark. Front Oncol 4, 262 (2014). 25325014 10.3389/fonc.2014.00262 PMC4179679

[b28] Singh S.R., Tan M. & Rameshwar P. Cancer metabolism: targeting metabolic pathways in cancer therapy. Cancer Lett 356, 147–148 (2015). 24956174 10.1016/j.canlet.2014.06.002

[b29] Boroughs L.K. & DeBerardinis R.J. Metabolic pathways promoting cancer cell survival and growth. Nat Cell Biol 17, 351–359 (2015). 25774832 10.1038/ncb3124 PMC4939711

[b30] Chen K.Y. *et al.* A metabolic signature of colon cancer initiating cells. Conf Proc IEEE Eng Med Biol Soc 2014, 4759–4762 (2014). 25571056 10.1109/EMBC.2014.6944688 PMC4302416

[b31] Witting M. *et al.* DI-ICR-FT-MS-based high-throughput deep metabotyping: a case study of the Caenorhabditis elegans-Pseudomonas aeruginosa infection model. Anal Bioanal Chem 407, 1059–1073 (2015). 25428456 10.1007/s00216-014-8331-5

[b32] Vlashi E. *et al.* Metabolic state of glioma stem cells and nontumorigenic cells. Proc Natl Acad Sci USA 108, 16062–16067 (2011). 21900605 10.1073/pnas.1106704108 PMC3179043

[b33] Lin S. *et al.* Hippocampal metabolomics using ultrahigh-resolution mass spectrometry reveals neuroinflammation from Alzheimer’s disease in CRND8 mice. Anal Bioanal Chem 405, 5105–5117 (2013). 23494273 10.1007/s00216-013-6825-1

[b34] Lin S. *et al.* Ultrahigh resolution mass spectrometry-based metabolic characterization reveals cerebellum as a disturbed region in two animal models. Talanta 118, 45–53 (2014). 24274269 10.1016/j.talanta.2013.09.019

