# Supplementary material for: Regulatory role of hexosamine biosynthetic pathway on hepatic cancer stem cell marker CD133 under low glucose conditions
Source: Sci Rep. 2016 Feb 16;6:21184. doi: 10.1038/srep21184 (PMC4754761; doi:10.1038/srep21184)
Supplement: Supplementary Information [file srep21184-s1.pdf]

Supplementary information

Regulatory role of hexosamine biosynthetic pathway on hepatic cancer stem cell marker CD133 under low glucose conditions

Shu-Hai Lin[1,+], Tengfei Liu[1,+], Xiaoyan Ming[2], Zhi Tang[1], Li Fu[3], Philippe Schmitt-Kopplin[4], Basem Kanawati[4], Xin-Yuan Guan[2,*], Zongwei Cai[1,*]

[1] State Key Laboratory of Environmental and Biological Analysis, Department of Chemistry, Hong Kong Baptist University, Hong Kong SAR;

[2] Department of Clinical Oncology, Li Ka Shing Faculty of Medicine, the University of Hong Kong, Hong Kong SAR;

[3] Shenzhen University Cancer Center, Shenzhen, 518060, China;

[4] Helmholtz-Zentrum Muenchen-German Research Center for Environmental Health, Research Unit of analytical Biogeochemistry, Ingolstaedter Landstrasse 1, D-85764 Oberschleißheim, Germany;

[+] These authors contributed equally to this work.

[*] Corresponding authors: zwcai@hkbu.edu.hk (Z.C.), xyguan@hku.hk (X.Y.G.)

Methods

Metabolite extraction

Cells were frozen at −80 °C until use, metabolites were extracted and proteins precipitated by adding 600 μL of cold acetone to the cell pellet (~ 1.0×10^6 cells for each replicate). Samples were vortex-mixed for 10 sec and submerged 1 min in liquid nitrogen, and then thawed (3-5 minutes) at room temperature and sonicated 3 min at 0 °C (icy water bath). This process was repeated three times and samples were stored at −20 °C for 1h. The pellet was removed by centrifuging at 8000 rpm for 10 min at 4 °C. The supernatant was transferred to a clean tube. Then, 400 μL of cold acetone/water (50: 50) was added to the pellet. Samples were vortex-mixed 10 sec, then submerged 1 min in liquid nitrogen and thawed (3-5 minutes) at room temperature. This process was repeated two times and samples were stored at −20 °C for 1h. The pellet was removed by centrifuging at 8 000 rpm for 10 min at 4 °C. The supernatant was pooled with the first extraction in the clean tube. Samples were dried under the gentle nitrogen stream and freeze-dried until complete dryness.

DI-ICR-FT-MS conditions

High-resolution mass spectra for molecular formula assignment were acquired on an ion cyclotron resonance-Fourier transform mass spectrometry (ICR-FT/MS, Solarix, Bruker Daltonics, Bremen, Germany) equipped with a 12-Tesla superconducting magnet and an Apollo II electrospray ion (ESI) source. The electric potential difference between the ESI needle and the counter electrode cone was 3500 V. The electric acceleration potential difference between the counter electrode cone and an additional installed metal cone was 500 V. Each sample was introduced into the ionization source at a flow rate of 2 μL min^{-1} by a microliter pump with a nebulizer gas pressure of 20 psi and a drying gas pressure of 15 psi (heated to 200 °C). All samples were measured three times in both negative and positive ion electrospray modes. To reduce systematic error associated with instrumental drift, samples were run in an order that alternated between CD133-positive and CD133-negative sample sets.

The instrument was calibrated based on cluster ions of 5 mg/L arginine in methanol. Calibration errors in the relevant mass range were always below 0.05 ppm and the standard deviation was always less than 10% by consecutive ten injections in negative ion mode. The spectra were

acquired with a time domain transient of two Mega words with a mass-to-charge ratio (*m/z*) range between 150 and 1,200 amu. This time domain transient length used for these measurements gave a mass resolving power of more than 190,000 at *m/z* =400. Three hundred scans were summed up in each acquisition. The ion accumulation time in the ion source was set to 100 milliseconds.

UPLC-QTOFMS procedure

A 5 μL aliquot of the supernatant was injected onto a ACQUITY UPLC BEH Amide column (1.7 μm, 2.1 × 100 mm, Waters). The mobile phase composition used was in 5% water containing 10 mM ammonium acetate and 0.1% formic acid in acetonitrile (A), and 50% water containing 10 mM ammonium acetate and 0.1% formic acid in acetonitrile (B). The sample separation is delivered at a flow rate of 200 μL/min as a linear gradient from 100% A for 2 min and decreased to 0% A over 13 min and held 4.5 min, then re-equilibrium in 0.5 min to 100% A and held 5 min for a total runtime of 25 min. The mass spectrometric measurement is performed on a MaXis UPLC/microTOF MS system in both positive and negative ion modes. Capillary voltage: −4500 V (negative) or 5000 V (positive); Dry temperature: 300 °C; Dry gas: 5.0 L/min. mass range is 50-1000 *m/z* in full scan mode.

The standard mixtures were inserted in sample running and used for metabolite identification. Standard mixture A consists of L-arginine, L-asparagine, L-glutamine, L-glutamate, L-histidine, L-leucine, L-serine, L-glycine, L-alanine, L-tryptophan and L-tyrosine (5 ppm for each standard); standard mixture B consists of L-isoleucine, L-methionine, L-proline, L-valine, L-lysine, L-aspartate, L-phenylalanine (5 ppm for each standard); standard mixture C consists of citrate, malate, succinate and fumarate (5 ppm for each standard). Moreover, we also used flow injection of the lock mass standards during sample running. This is a useful method to maintain the accurate mass measurement in UPLC-QTOFMS analysis.

Figure captions

Figure S1. The overall mass spectra of $CD133^+$ and $CD133^-$ cells, respectively, in the whole mass range *m/z* 150-1,000 of positive ion mode.

Figure S2. Real-time PCR analysis of mRNA expression of SOX2 under high and low glucose conditions (left panel) and Western blot of SOX2 under low glucose condition (right panel) of PLC8024 cells.

Figure S3. GlcNAc restores the CD133-positive subsets within Huh7 and Hep3b that exposed to glycolytic inhibition. Huh7 and Hep3B cells were cultured in normal medium with 20 mM glycolytic inhibitor 2-DG in presence of 10 mM GlcNAc or not for 24 h. The proportions of CD133+ subpopulation cells were determined by flow-cytometry.

Figure S1.

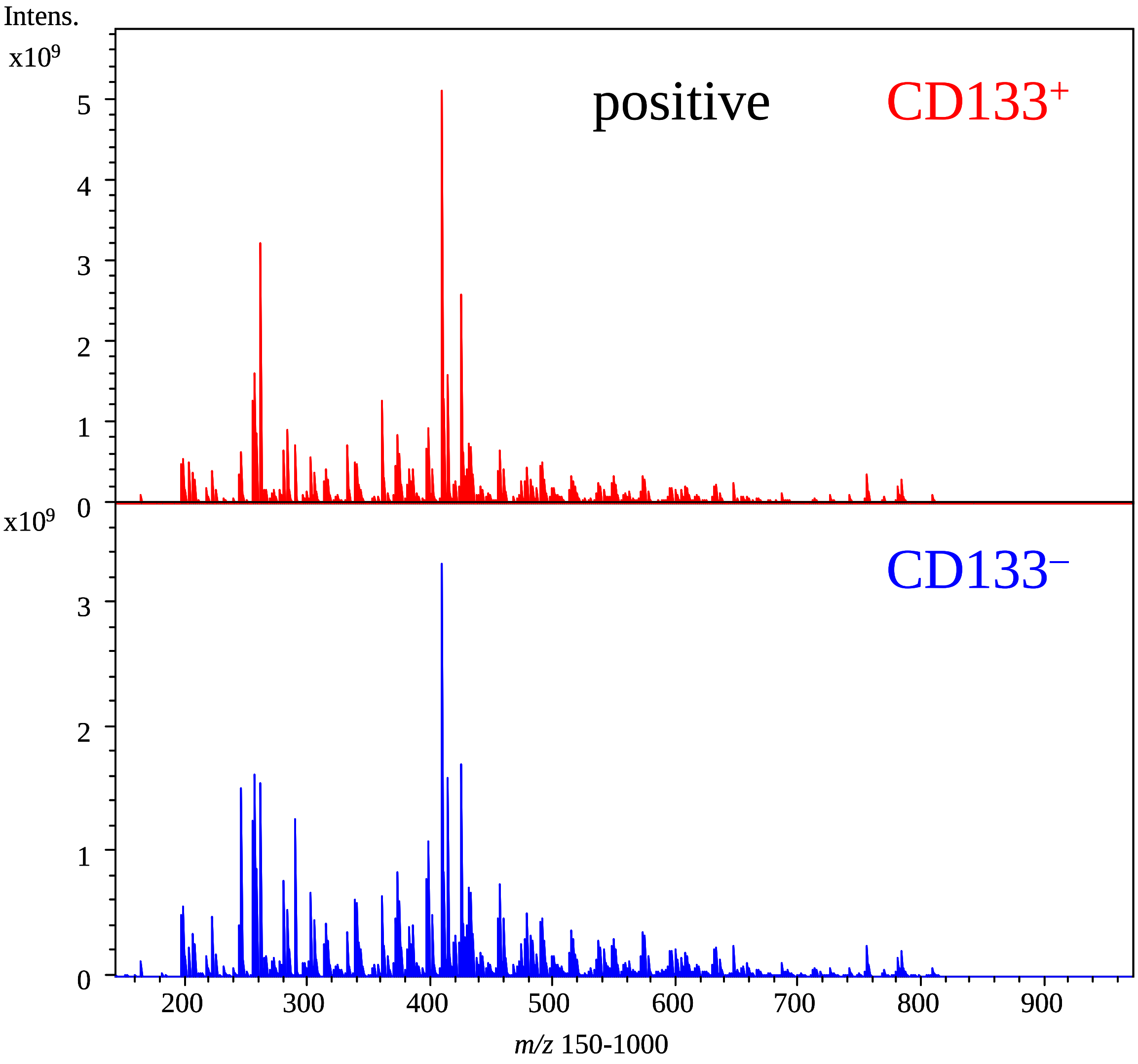
Intens.
x10^9
5
4
3
2
1
0
positive
CD133+
x10^9
3
2
1
0
CD133–
200
300
400
500
600
700
800
900
m/z 150-1000

Figure S2.

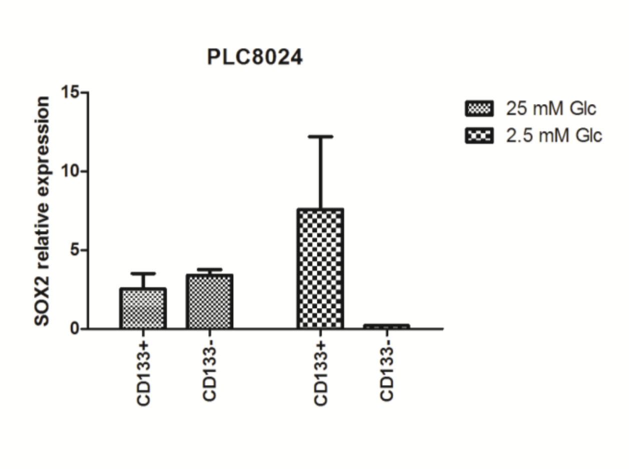
PLC8024
SOX2 relative expression
15
10
5
0
25 mM Glc
2.5 mM Glc
CD133+
CD133-
CD133+
CD133-

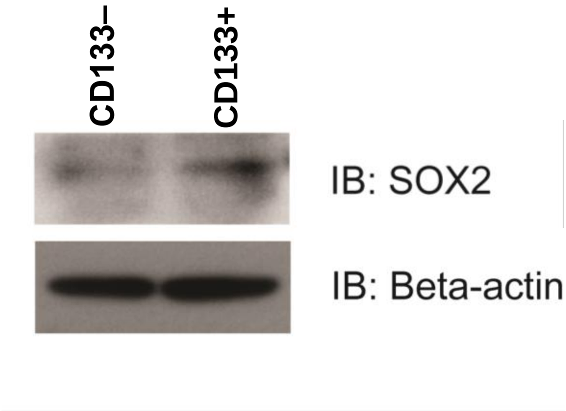
CD133–
CD133+
IB: SOX2
IB: Beta-actin

Figure S3.

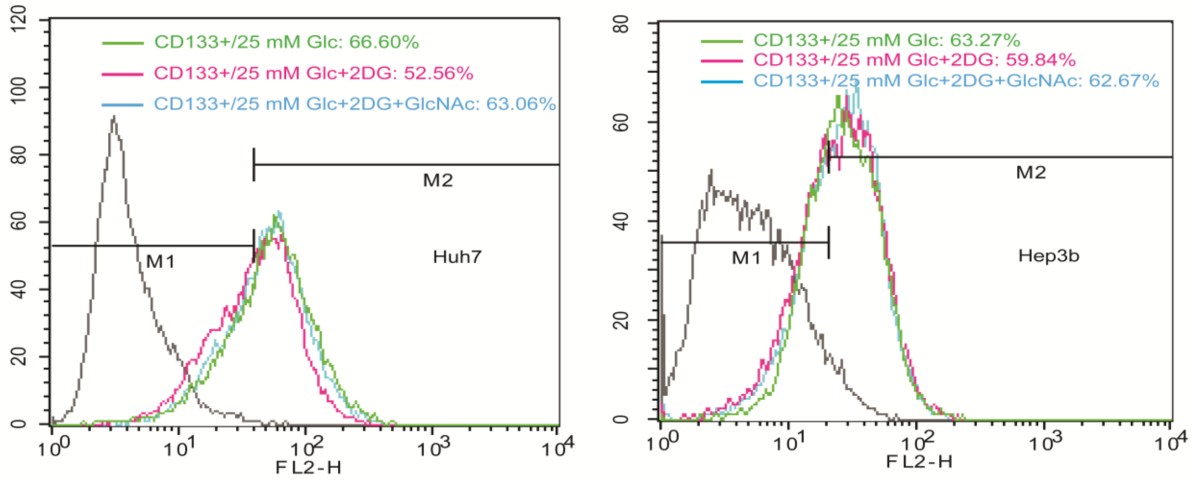

CD133+/25 mM Glc: 66.60%
CD133+/25 mM Glc+2DG: 52.56%
CD133+/25 mM Glc+2DG+GlcNAc: 63.06%
M1
M2
Huh7
FL2-H
CD133+/25 mM Glc: 63.27%
CD133+/25 mM Glc+2DG: 59.84%
CD133+/25 mM Glc+2DG+GlcNAc: 62.67%
M1
M2
Hep3b
FL2-H

Table S1

The primers used for quantitative real-time PCR analysis

Gene	Primer sequences
GLUT1-F	5’-GGCCAAGAGTGTGCTAAAGAA-3’
GLUT1-R	5’-ACAGCGTTGATGCCAGACAG-3’
GLUT2-F	5’-GGGCAATTATGATCTGTGGCA-3’
GLUT2-R	5’-TTCTGCTCACTCGATGCTTCT-3’
GAPDH-F	5’-GGAGCGAGATCCCTCCAAAAT-3’
GAPDH-R	5’-GGCTGTTGTCATACTTCTCATGG-3’
PGAM1-F	5’-GCTAATCCCAGTCGGTGCC-3’
PGAM1-R	5’-ATAGCCAGCATCTCGTAGCG-3’
PKM2-F	5’- ATAACGCCTACATGGAAAAGTGT-3’
PKM2-R	5’-TAAGCCCATCATCCACGTAGA-3’
LDHA-F	5’- ATGGCAACTCTAAAGGATCAGC-3’
LDHA-R	5’-CCAACCCCAACAACTGTAATCT-3’
PFKL-F	5’- GCTGGGCGGCACTATCATT-3’
PFKL-R	5’-TCAGGTGCGAGTAGGTCCG-3’
SOX2-F	5’-GGGAAATGGGAGGGGTGAAAAGAGG-3’
SOX2-R	5’- TTGCGTGAGTGTGGATGGGATTGGTG-3’
18s-qF	5’-CTCTTAGCTGAGTGTCCCGC-3’
18s-qR	5’-CTGATCGTCTTCGAACCTCC-3’
